# Systematic Review of Large Language Models and Natural Language Processing in Stroke Care: Applications, Challenges, and Future Directions

**DOI:** 10.1161/SVIN.125.002261

**Published:** 2026-05-05

**Authors:** Kaue Tartarotti Nepomuceno Duarte, Abhijot Singh Sidhu, Maya Bakshi, Murilo Costa de Barros, Brij Karmur, Taha Aslan, Donghao Zhang, Mohamed AlShamrani, Wu Qiu, Bijoy K. Menon

**Affiliations:** Departments of Clinical Neuroscience and Radiology, Hotchkiss Brain Institute, Cummings School of Medicine (K.T.N.D., B.K., B.K.M.), University of Calgary, Alberta, Canada.; Graduate Program in Biomedical Engineering, Department of Radiology (A.S.S.), University of Calgary, Alberta, Canada.; Calgary Stroke Program, Department of Clinical Neurosciences, Foothills Medical Centre (T.A., M.A., B.K.M.), University of Calgary, Alberta, Canada.; Seaman Family MR Research Centre, Foothills Medical Centre, Calgary, Canada (A.S.S.).; Department of Physiology, McGill University, Montreal, Quebec, Canada (M.B.).; School of Technology, University of Campinas, Limeira, Brazil (M.C.B.).; College of Life Science and Technology, Huazhong University of Science and Technology, Wuhan, China (D.Z., W.Q.).

**Keywords:** artificial intelligence, machine learning, natural language processing, stroke, systematic review

## Abstract

Stroke, a leading cause of mortality, manifests as ischemic (87%) or hemorrhagic (13%), demanding rapid intervention to mitigate irreversible damage. Despite advances in artificial intelligence, systematic reviews addressing the integration of large language models and natural language processing into clinical stroke care remain limited. Such a review is critical given large language models’ potential to overcome traditional natural language processing limitations, thereby enhancing risk prediction and decision support. We proposed a systematic review aimed to comprehensively analyze applications in stroke care. After searching 6 databases, 2991 records were screened, yielding 65 eligible studies. Results were structured quantitatively (impact factor trends, publication distribution) and qualitatively across 4 dimensions: Study Purposes (eg, risk modeling, decision support), Data Sets/Key Findings, Limitations, and Future Directions. Large language models demonstrated strong capabilities in automated data extraction from clinical notes (accuracies of 93.5%–95.1%) and report summarization. However, majority of studies (94%) lacked external validation. Most were limited by single-center, retrospective designs (62%) and used private data sets (85%), raising concerns about generalizability. Common failure modes included model hallucinations, performance degradation on external data, and infrastructural barriers to clinical integration. Future efforts must prioritize multicenter, prospective validation (82% of studies) to ensure model robustness and generalizability across diverse populations. Pathways for clinical translation include developing interpretability techniques to build clinician trust. Technical refinements for hallucination mitigation (98% of studies) and real-time integration of multimodal data are necessary to enhance predictive power. Addressing data heterogeneity and ethical concerns remains as gaps. This review highlights the potential of large language models in stroke care, encompassing tasks from risk prediction to workflow automation. Realizing this potential requires a shift from proof-of-concept studies to rigorously validated, clinically integrated systems. The field demands scalable, equitable, and transparent artificial intelligence solutions that are codeveloped with clinicians. These are needed to overcome existing methodological and translational barriers.

## Graphical abstract

**Figure:**
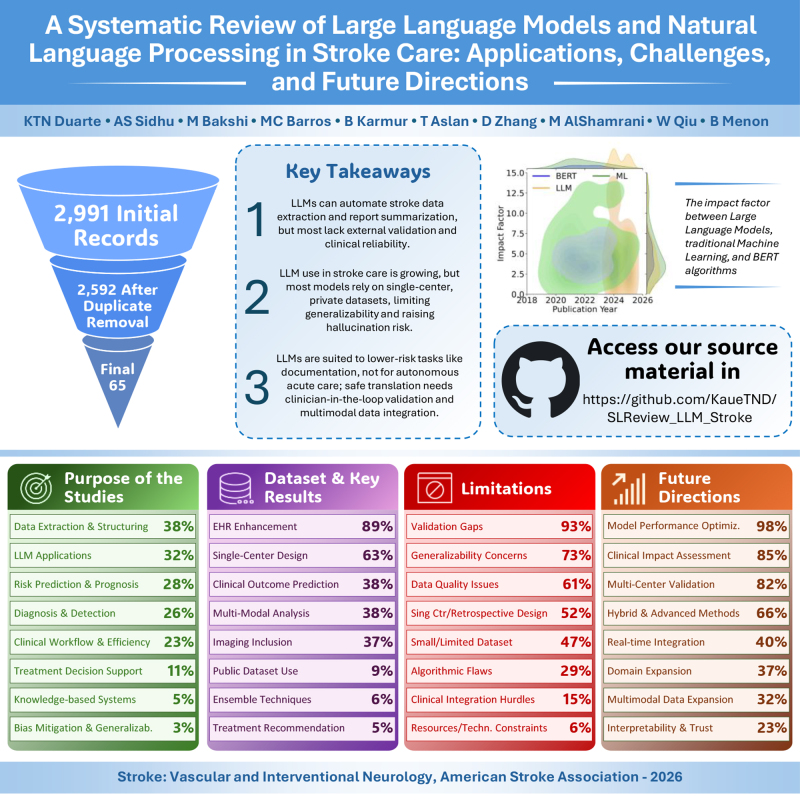

Stroke is a leading cause of mortality and long-term disability worldwide.^1^ In clinical practice, stroke care is defined by extreme time sensitivity (time is brain), reliance on high-quality neurological deficit documentation, and the central role of neuroimaging in diagnosis and treatment selection.^1,2^ These characteristics make stroke a uniquely high-stakes domain for language-based clinical decision support, where errors or delays may translate into preventable disability.

Beyond the hyperacute phase, stroke management involves complex longitudinal care, including secondary prevention, rehabilitation, and monitoring for complications, such as depression, cognitive impairment, and functional decline. Across this continuum, key clinical information is frequently embedded in unstructured narrative data, including emergency department notes, neurological examinations, radiology reports, and discharge summaries, often generated under significant time pressure and across multiple care settings.^3^

Artificial intelligence (AI) techniques have shown promising results for stroke research, excelling in tasks such as automated lesion detection and segmentation,^4,5^ occlusion,^6,7^ reconstruction of perfusion maps,^8,9^ predicting hemorrhagic transformation risk,^10,11^ and quantification of collateral circulation.^12,13^ Among the machine learning (ML) types, natural language processing (NLP) is responsible for extracting structured insight patterns from unstructured data, such as clinical notes, radiology reports, and electronic health records (EHR). However, NLP techniques often struggle with integrating longitudinal data, generalizability, contextual differences across data sets, and reliance on rule-based approaches. To circumvent this issue, large language models (LLMs) have been proposed to not only account for different clinical contexts but also to offer richer representations for interpreting clinical data, and more importantly, facilitate stroke care. In stroke, this limitation is clinically important because the most actionable details often appear in narrative form (eg, National Institutes of Health Stroke Scale [NIHSS] descriptors, aphasia-related uncertainty, thrombectomy eligibility language, contraindications, and radiology impressions).^14^ LLMs may offer incremental value over traditional NLP by synthesizing longitudinal narrative notes and reports into structured, clinically interpretable summaries that can support audit, documentation, and (with safeguards) decision support.

LLMs are rapidly transforming how information is processed across society, from education to healthcare. In medicine, they overcome many traditional ML and NLP limitations through contextual reasoning, multimodal data integration, and adaptability via in-context learning and retrieval-augmented generation. For stroke care, these capabilities can enhance risk assessment, clinical decision support, and equitable access to expertise—even in resource-limited settings. Yet, current applications remain fragmented. This systematic review maps existing work, evaluates methodological rigor, and outlines key challenges and future directions, including bias mitigation, interpretability, and data privacy with the use of LLM for stroke research and care. We reported the notations in machine learning (Table) to facilitate the reading of this work.

**Table.**
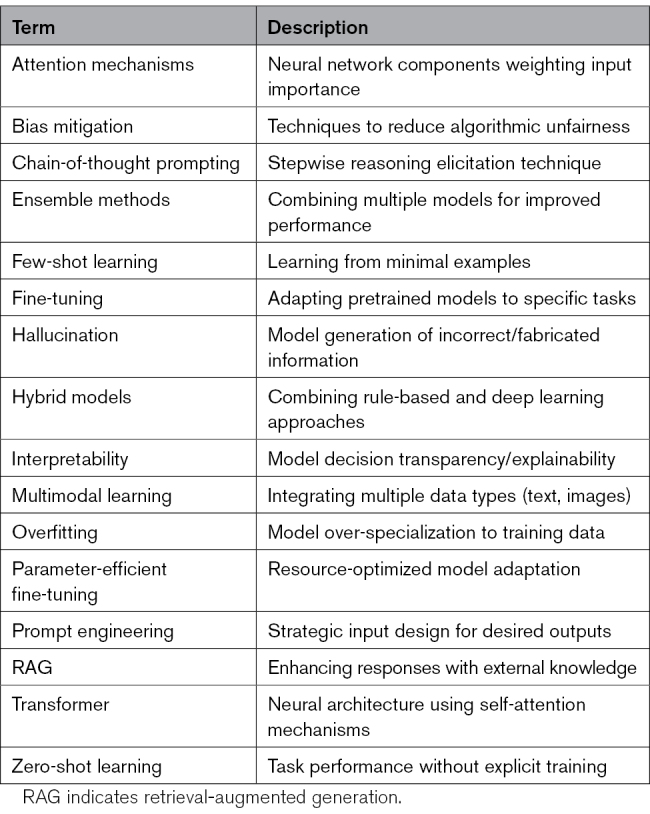
Notation of Machine Learning Terms

## Systematic Review Protocol

This review followed the Preferred Reporting Items for Systematic Reviews and Meta-Analyses guidelines and adhered to established reproducibility standards.^15^ The protocol was prospectively developed and made publicly available on GitHub (GitHub repository: https://github.com/KaueTND/SLReview_LLM_Stroke).

### Objectives

The primary objective was to identify, synthesize, and critically evaluate studies that applied LLM and NLP techniques to any aspect of stroke care—diagnosis, risk prediction, clinical decision support, audit automation, or workflow optimization. Although this review primarily focuses on LLM, the search strategy intentionally included broader terms related to NLP. This approach ensures comprehensive capture of both traditional NLP and transformer-based studies, recognizing that LLMs have evolved directly from earlier NLP frameworks. Secondary objectives included the following: (1) examining temporal and methodological trends in the use of LLM; (2) assessing variability in LLM architecture used, validation rigor, and interpretability; and (3) highlighting gaps and opportunities for clinical translation. Analyze focal points in model development and deployment.

### Research Questions (Population, Intervention, Comparison, Outcome Framework)

We used the following Population, Intervention, Comparison, Outcome framework below. The Population, Intervention, Comparison, Outcome framework was adapted to capture both conventional NLP and emerging LLM studies, reflecting the progressive evolution of language-based AI methods in stroke research and care.

- Population: Patients with ischemic or hemorrhagic stroke (cerebrovascular accident [CVA] or stroke) whose diagnosis, management, or outcomes were analyzed using text-based clinical data such as EHRs, clinical notes, or radiology reports.
- Intervention: Application of language-based AI methods, including both conventional NLP techniques (eg, rule-based systems, bidirectional encoder representations from transformers [BERT]-family models) and modern LLM (eg, generative pretrained transformer [GPT], Large Language Model Meta AI [LLaMA], transformer-based architectures), for tasks such as risk prediction, report summarization, decision support, or workflow optimization.
- Comparison: Traditional machine learning, non-AI, or manual approaches, if available. Where no direct comparator was reported, descriptive comparisons were made across NLP and LLM categories to assess methodological and performance evolution.
- Outcome: Model performance (accuracy, area under the curve [AUC], F1 score [harmonic mean of precision/recall], sensitivity/specificity), validation rigor (internal/external), interpretability, scalability, and barriers to clinical adoption, including generalizability, bias, and ethical or regulatory considerations.

### Search Strategy

A comprehensive electronic search was conducted in 6 databases: PubMed, Web of Science, Institute of Electrical and Electronics Engineers (IEEE) Xplore, Elsevier (Scopus), SpringerLink, and Association for Computing Machinery (ACM) Digital Library. The final search was completed in May 2025. Search terms combined controlled vocabulary (MeSH) and free-text keywords for stroke and language-model/NLP concepts. A 2-tier structure of “controller” and “variant” terms was used:

- Controller (primary) terms: “stroke,” “cerebrovascular accident,” “LLM,” “natural language processing,” “transformer model.”
- Variant (secondary) terms: “ischemic stroke,” “hemorrhagic stroke,” “GPT,” “BERT,” “clinical NLP,” “report generation.”

The boolean syntax was customized per database:

- Web of Science: TS=(“stroke” OR “cerebrovascular accident” OR CVA OR “ischemic stroke” OR “hemorrhagic stroke”) AND TS=(“large language model” OR LLM OR GPT OR BERT OR “natural language processing” OR “transformer model”).
- PubMed: (“Stroke”[MeSH] OR stroke[tiab] OR “cerebrovascular accident”[tiab] OR CVA[tiab] OR “ischemic stroke”[tiab] OR “hemorrhagic stroke”[tiab]) AND (“large language model”[tiab] OR LLM[tiab] OR GPT[tiab] OR BERT[tiab] OR “transformer model”[tiab] OR “natural language processing”[tiab]).
- IEEE Xplore, Elsevier, Springer, ACM: (“stroke” OR “cerebrovascular accident” OR CVA OR “ischemic stroke” OR “hemorrhagic stroke”) AND (“large language model” OR LLM OR GPT OR BERT OR “natural language processing” OR “transformer model”).

No date restrictions were applied, but only English language papers published in January 2020 or later were retained to capture the modern LLM era. Search results were exported as comma separated valued files, including citation metadata and abstracts, for screening.

### Study Selection

Two independent reviewers (R1 and R2) screened all titles and abstracts using predefined criteria.

- Inclusion criteria: (1) LLM/NLP methods applied to stroke diagnosis, prognosis, or clinical decision support; (2) original peer-reviewed research; (3) English language.
- Exclusion criteria: Reviews, nonstroke applications, non-AI methods, and studies without quantitative or qualitative outcomes.

Disagreements were resolved by consensus or adjudication by a third reviewer (R3). The inter-reviewer agreement was *K*=0.63 (moderate). A 3-stage filtering pipeline was implemented:

1. title/abstract screening;
2. full-text eligibility assessment; and
3. consensus resolution and data extraction.

**Figure 1.**
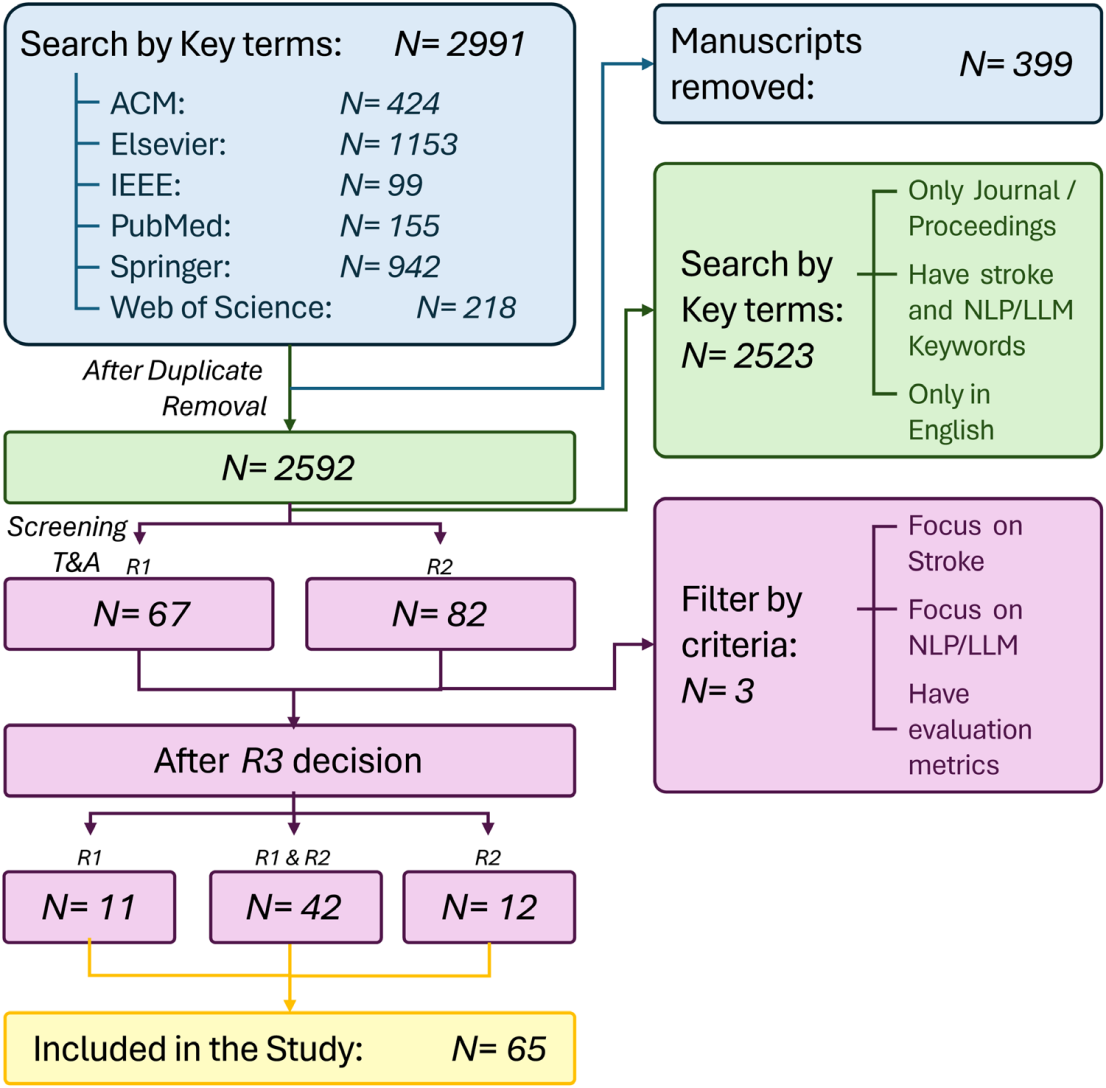
**PRISMA workflow (Preferred Reporting Items for Systematic Reviews and Meta-Analyses) for systematic review of large language models (LLMs) in stroke research.** ACM indicates Association for Computing Machinery; IEEE, Institute of Electrical and Electronics Engineers; and NLP, natural language processing.

The full Preferred Reporting Items for Systematic Reviews and Meta-Analyses workflow is summarized in Figure 1.

### Data Extraction and Quality Assessment

A standardized template was used to capture:

- study metadata (year, journal, country, impact factor);
- model characteristics (architecture, training approach, data modality);
- data set type and size;
- evaluation metrics (accuracy, AUC, F1, sensitivity/specificity); and
- reported limitations and clinical implications.

Quantitative results (eg, impact factor trends, publication distribution) were summarized descriptively; qualitative themes were analyzed across 4 analytic dimensions: Purpose, Data Set and Findings, Limitations, and Future Directions. Figure 1 outlines the Preferred Reporting Items for Systematic Reviews and Meta-Analyses workflow. Initial searches yielded 2991 papers, reduced to 66 after rigorous filtering. The protocol and data sets are publicly available on GitHub (GitHub repository: https://github.com/KaueTND/SLReview_LLM_Stroke).

## Results

Initial searches yielded 2991 records, of which 2592 remained after automatic and manual duplicate removal. Following title and abstract screening by R1 and R2, 67 and 82 studies were retained, respectively. R3 resolved discrepancies, producing a final inclusion set of 65 eligible papers. The complete list of included studies, database-specific queries, and extracted data sets is available in the accompanying GitHub repository. The inter-reviewer agreement between R1 and R2 reached 63%. A third independent reviewer (ie, R3) was responsible for resolving the discrepancies between R1 and R2, resulting in 11 papers from the R1’s list and 12 from the R2’s list (Figure 1). Ultimately, a final list of 65 papers was selected for this study.

### Quantitative Evaluation of Retrieved Papers

**Figure 2.**
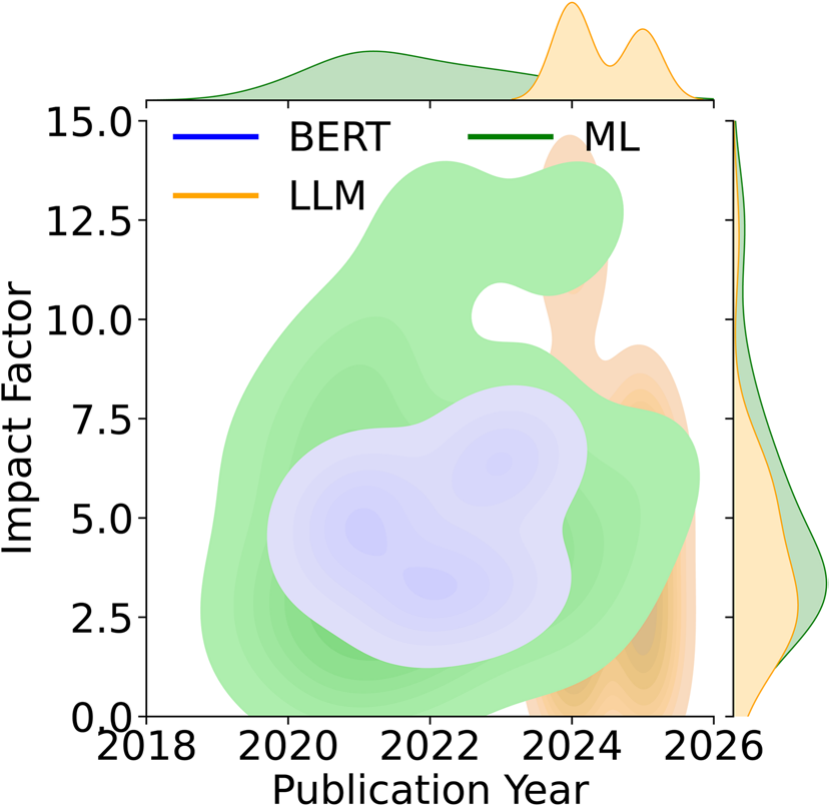
Heatmap illustrating the use of large language model (LLM), traditional machine learning (ML), and bidirectional encoder representations from transformers (BERT) algorithms over time (years on the *x* axis), with impact factor values on the *y* axis.

**Figure 3.**
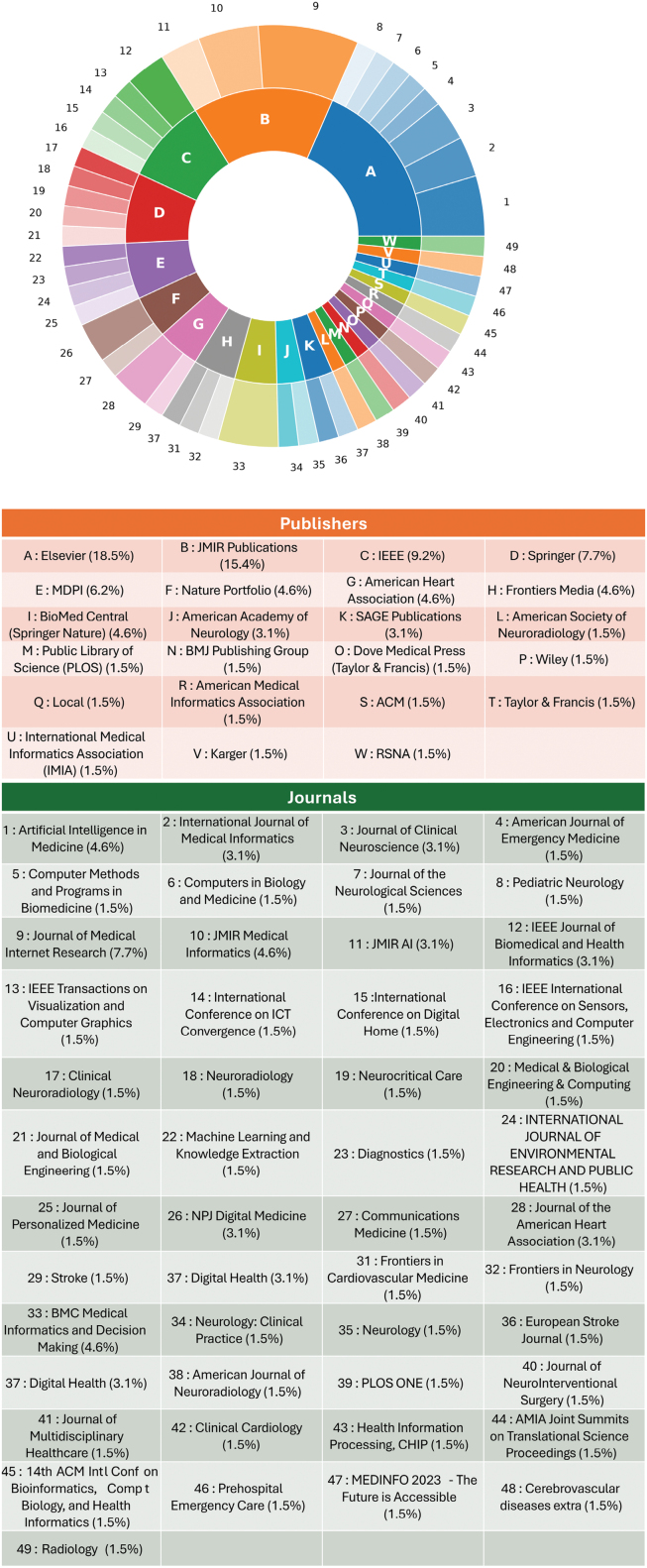
**Pie chart showing the number of journal publications per publisher.** Inner-circle letters denote publisher abbreviations; outer-circle numbers correspond to specific journal names. ACM indicates Association for Computing Machinery; AMIA, American Medical Informatics Association; BMC, BioMed Central; BMJ, British Medical Journal; CHIP, Chinese Journal of Health Informatics and Management; IEEE, Institute of Electrical and Electronics Engineers; JMIR, Journal of Medical Internet Research; MDPI, Multidisciplinary Digital Publishing Institute; NPJ, Nature Partner Journals; and RSNA, Radiological Society of North America.

To analyze the use and impact of each article reviewed in this work, we grouped them based on: (1) the impact factor of the journal, and (2) the name and style of the journals. First, we examined the impact factor stratified by technique (Figure 2). In this review, we divided the technique by model complexity, categorizing the computational techniques into 3 distinct groups: Traditional Machine Learning(encompassing feature-engineered models like Random Forest, Extreme Gradient Boosting algorithm, and support vector machines for structured data analysis and rule-based NLP), BERT-based models(leveraging domain-specific transformer architectures pretrained on biomedical data for deep semantic understanding of clinical text), and LLM (utilizing both general-purpose and medically tuned foundational models for complex, context-aware tasks like zero-shot information extraction and clinical reasoning). Although NLP approaches have consistently demonstrated higher impact factors over time, LLM publications have shown rapid growth in recent years. Within just 2 years, the volume of LLM publications has reached nearly half of that of traditional ML and BERT publications, reflecting the accelerated translation of research into practice. Figure 3 illustrates the distribution of publications across journals and conference proceedings, revealing a predominance of AI research focused on neurology and radiology.

### Quality Evaluation of Retrieved Papers

To systematically characterize the research landscape, we developed a framework of analytic themes through a structured, inductive process. The 4 primary dimensions—Purpose, Data Set and Key Results, Limitations, and Future Directions—were selected as they represent the core, universal components of empirical research papers, enabling a consistent cross-study comparison.

The subtopics (A–H) within each dimension were not predefined. Instead, they were derived empirically via a systematic content analysis of the reviewed articles. The process involved the following steps: Each dimension contained 8 subthemes (A–H) defining the main objectives (eg, risk prediction, decision support), data characteristics (eg, multimodal versus single-center), methodological gaps (eg, validation, bias), and proposed next steps (eg, multicenter validation, interpretability).

The presence of each subtheme in a given article was assessed using a binary scoring system (Yes/No). A score of 1 (Yes) was assigned if the subtheme was a central focus of the article’s contribution (for Purpose and Data Set and Key Results) or a primary stated concern (for Limitations and Future Directions). A score of 0 (No) was assigned otherwise.

**Figure 4.**
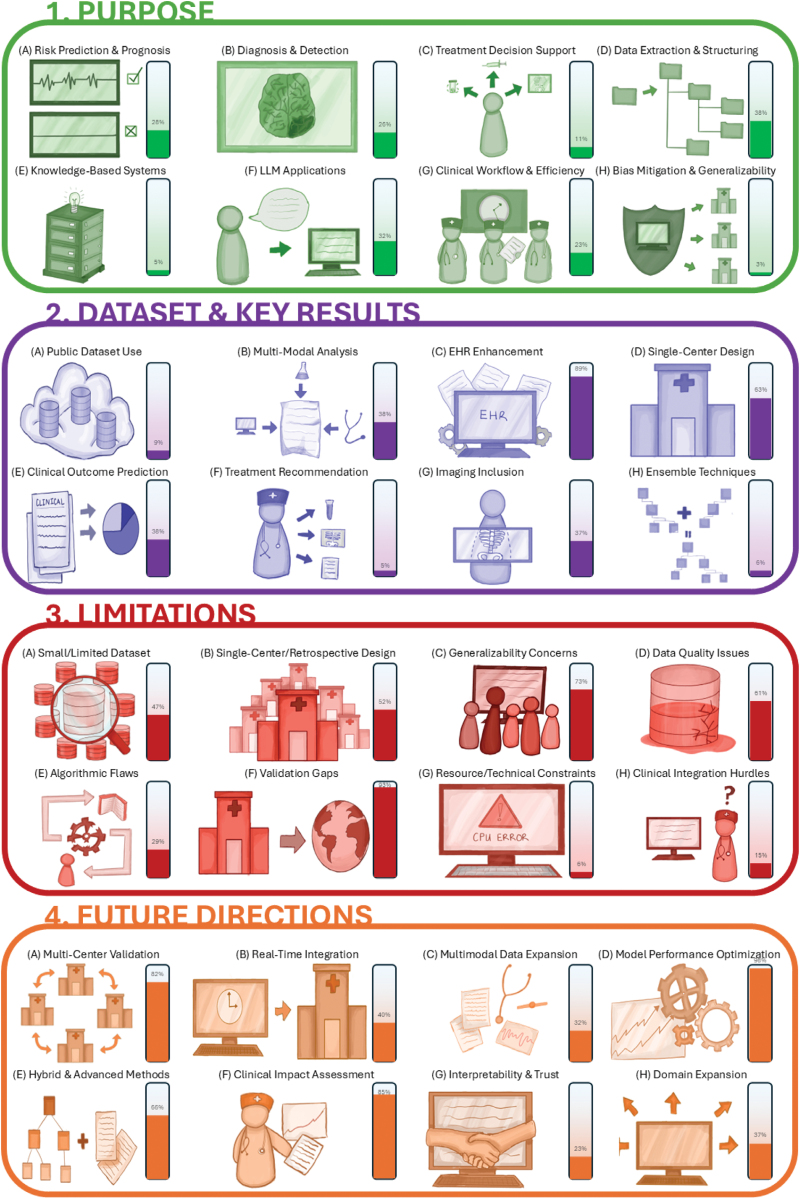
**Illustrative view of term (grouped by analytic dimension) and their occurrence percentages (shown in the lateral bars).** EHR indicates electronic health record; and LLM indicates large language model.

**Figure 5.**
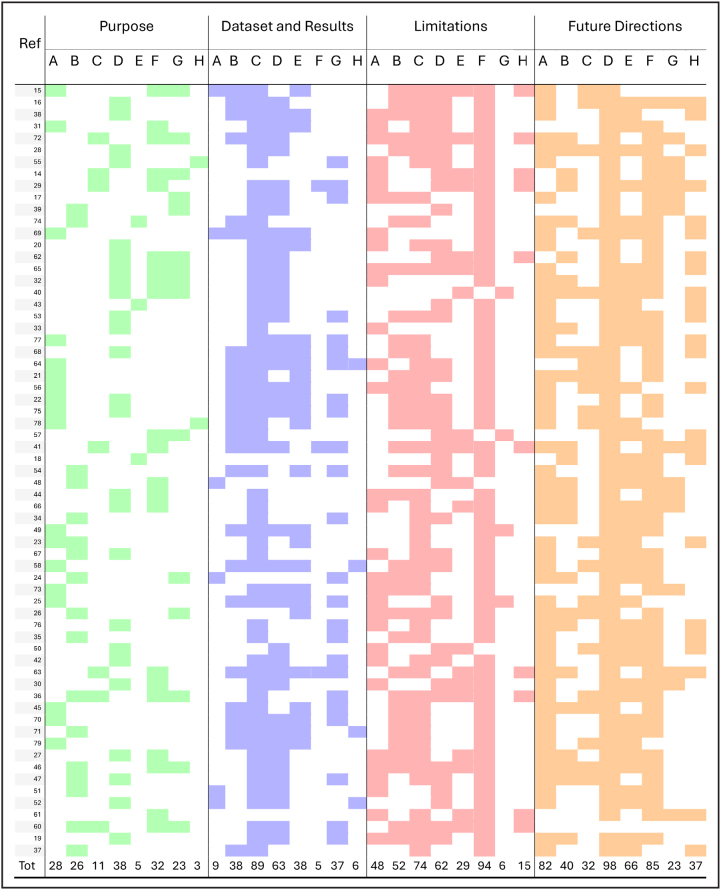
**Results of a systematic content analysis where each study (row) was evaluated for the presence of predefined subthemes (columns—subtheme definitions [A through H] are provided in section Quality Evaluation of Retrieved Papers).** A colored cell indicates a Yes (the subtheme was a central focus of the study), while a white cell indicates a No. The Total row reports the total percentage of the presence of each subtheme (Purpose, Data Set and Key Results, Limitations, Future Directions). Color is used for the visual distinction of the 4 dimensions only.

The resulting data, visualized in Figures 4 and 5, represents the frequency count—the number of papers for which each subtheme was marked Yes. This allows for a quantitative overview of prevailing research trends, common methodological challenges, and collective future ambitions within the domain of AI and stroke care.

- Purpose:
  (A) Risk prediction and prognosis: Models estimating stroke risk, recurrence probability, functional outcomes, or mortality rates.
  (B) Diagnosis and detection: Techniques for accurate identification of stroke events, subtypes, or related conditions from clinical data.
  (C) Treatment decision support: Tools aiding selection of therapies (eg, thrombolysis, thrombectomy) based on patient-specific features.
  (D) Data extraction and structuring: Automated parsing of unstructured text (eg, radiology reports, EHR notes) into standardized data fields.
  (E) Knowledge-based systems: Ontology- or rule-based frameworks leveraging medical vocabularies and logic for inference beyond simple pattern matching.
  (F) LLM applications: Use of LLMs (eg, GPT, LLaMA) for tasks like report generation, summarization, or clinical question and answer (Q&A).
  (G) Clinical workflow and efficiency: Solutions targeting process optimization, reducing time to diagnosis/treatment, and minimizing clinician workload.
  (H) Bias mitigation and generalizability: Strategies to detect, reduce, or account for data set/model biases and ensure broad applicability.
- Data Set and Key Results:
  (A) Public data set use: Utilization of open-source stroke-related data sets for training and benchmarking.
  (B) Multimodal analysis: Integration of different data types (*eg,* imaging, lab values, clinical text) for richer modeling.
  (C) EHR enhancement: Augmenting EHRs with derived features or annotations.
  (D) Single-center design: Studies relying on data from a single institution without external cohorts.
  (E) Clinical outcome prediction: Performance metrics for forecasting patient outcomes poststroke.
  (F) Treatment recommendation: Evaluation of algorithms recommending optimal therapy pathways.
  (G) Imaging inclusion: Incorporation of computed tomography, magnetic resonance imaging, or ultrasound scans in model inputs.
  (H) Ensemble techniques: Combining multiple algorithms or models to improve robustness and accuracy.
- Limitations:
  (A) Small/limited data set: Insufficient sample sizes or narrow patient demographics.
  (B) Single-center/retrospective design: Lack of prospective and multicenter validation affecting reliability.
  (C) Generalizability concerns: Challenges transferring models across populations, languages, or settings.
  (D) Data quality issues: Missing, inconsistent, or noisy data compromising model training.
  (E) Algorithmic flaws: Model-specific errors such as hallucinations, overfitting, or lack of contextual reasoning.
  (F) Validation gaps: Absence of external benchmarking or real-world deployment studies.
  (G) Resource/technical constraints: High computational demands or limited modality support.
  (H) Clinical integration hurdles: Obstacles in embedding algorithms into everyday practice, including workflow and ethical concerns.
- Future Directions:
  (A) Multicenter validation: Expanding evaluation across diverse institutions to verify robustness.
  (B) Real-time integration: Embedding models directly into clinical systems for point-of-care decision support.
  (C) Multimodal data expansion: Incorporating wearable, genetic, and broader sensor-derived data streams.
  (D) Model performance optimization: Techniques to reduce hallucinations, refine prompts, and handle imbalanced data.
  (E) Hybrid and advanced methods: Combining rule-based systems with ML/LLMs and exploring novel architectures.
  (F) Clinical impact assessment: Rigorous studies measuring outcomes, cost-effectiveness, and workflow benefits.
  (G) Interpretability and trust: Developing explainable models with transparency and bias auditing tools.
  (H) Domain expansion: Applying stroke AI methods to new diseases, languages, and healthcare contexts.

### Motivations and Objectives of NLP and LLM in Stroke Care

From a clinical perspective, the distinction between traditional NLP and LLM approaches lies primarily in the level of contextual reasoning they can support. For example, conventional NLP methods may extract evidence of atrial fibrillation from discharge summaries or problem lists, whereas LLM-based systems can synthesize cardiology consultation notes, echocardiography reports, and longitudinal clinic documentation to generate a structured cardioembolic risk profile for the treating clinician. This shift from isolated information extraction to integrative clinical reasoning represents a key conceptual advance for stroke-related applications.

#### Risk Assessment and Prediction

Across included studies, AI methods were widely applied for personalized stroke risk assessment (28%) and clinical decision support. Several tools used chatbot interfaces to communicate individualized risk and guide treatment decisions, including thrombectomy eligibility and adherence to clinical guidelines.^16–19^

Models analyzing multimodal data—such as EHRs, imaging text, and longitudinal clinical information—used algorithms including the Extreme Gradient Boosting algorithm, attention mechanisms, and transformer-based architecture to predict outcomes such as readmission, functional recovery, and mortality risk.^20–27^

#### Treatment Decision Support

Several studies applied AI to optimize therapeutic decision-making in acute stroke care. LLM- and NLP-based models were utilized to determine eligibility for thrombectomy or thrombolysis, assess procedure contraindications, and recommend treatment pathways, using data from radiology reports and NIHSS scores.^17–19,28–30^ Some systems use synthetic notes to screen for thrombolysis side effects or apply reinforcement-learning algorithms to adjust local practice parameters and improve alignment with clinical guidelines.

#### Audit and Information Extraction

Automation of data extraction from unstructured clinical text, including radiology reports, discharge summaries, and procedural records, was a frequent application. Rule-based NLP pipelines, traditional classifiers, and hybrid models integrating BERT or active learning components have achieved high sensitivity in detecting key clinical variables.^31–37^

LLM-based approaches further enhanced extraction accuracy for stroke audit variables, rehabilitation monitoring, and protocol compliance, while reducing manual workload in resource-limited settings.^29,32,38–42^

#### Multilingual and Imaging Integration

Several studies incorporated multilingual processing and multimodal fusion of imaging and clinical text to improve model generalizability.

LLM architectures using chain-of-thought prompting, low-rank adaptation fine-tuning, and JSON (JavaScript Object Notation [data format])–based optimization were applied to combine textual and imaging data for lesion localization, subtype classification, and biomarker detection.^19,34,43–47^

Approaches that integrate radiology reports with computed tomography or magnetic resonance imaging features outperformed single-modality baselines in identifying large-vessel occlusions and etiologic subtypes.^22,26,45,47–52^

#### Ensemble and Hybrid Models

Multiple studies implemented ensemble learning or hybrid architectures that combined traditional ML algorithms (eg, Extreme Gradient Boosting algorithm, support vector machine) with deep learning or transformer components to enhance predictive performance. These approaches improved the classification of stroke cause, readmission risk, and atrial fibrillation detection by merging structured and unstructured EHR data.^24,27,48^ Ontology-based systems were also developed to standardize terminology and enable semantic query translation for stroke-related physical-examination data.^53,54^

#### Bias Mitigation and Generalizability

Efforts to address bias and generalizability were limited but emerging. Several studies have attempted to reduce sampling or institutional bias through synthetic data augmentation, external validation, or rule-based feature selection.^14,29,55–59^ Others assessed chatbot reliability for emergency care guidance across various domains and languages, highlighting variability in accuracy and consistency of advice.^45,60^ Overall, bias-mitigation strategies were infrequent, and most models remained single-center and language-restricted.

The use of bias mitigation and generalizable solutions address the data quality challenges. The goal is to design cohort groups while minimizing sampling bias, perform internal or external validation across different population groups, fill missing data with NLP, use synthetic data testing to quantify bias, and evaluate information across different institutions.^14,29,57,58^ Adopting rule-based and heuristic NLP models prioritizes interpretability by imputing scores via feature selection and combining rule-based pipelines with ML for phenotyping.^55,56,59^ Generative chatbots in emergency stroke care support have been evaluated for reliability and safety, comparing performance across domains such as accuracy and emergency advice variability, as well as fine-tuning models to integrate multiple modalities and improve baseline classification and screening capacities.

### Evaluation of Key Findings and Research Outcomes

#### Data Characteristics and Size

For stroke care, both public and private data sets are adopted for model development. Public data sets like Medical Information Mart for Intensive Care III data set and Mass General Brigham EHRs enable multisite validation, while proprietary (private) data sets normally provide more context-specific and single-site knowledge. Public data sets were adopted in 15% of the studies, primarily for validation, with the Medical Information Mart for Intensive Care III data set being the most dominant. In contrast, private data sets comprised 85% of the studies, with cohorts averaging around 1363 patients. Whereas multisite private data sets reached up to 100 000 patients, public data sets such as the Medical Information Mart for Intensive Care III data set and the United Kingdom Clinical Practice Research Datalink (UK CPRD) were used to analyze cohorts exceeding 50 000 patients, providing a contrast with the more common single-site studies, which often involved <1000 patients. Data imbalance, a common challenge in machine learning where one class of data significantly outnumbers others, was prevalent in the majority of studies (92%). This was evident in scenarios such as thrombectomy cases, which represented as low as 1.4% of a data set, and severe strokes, comprising only 5.4% of readmission cohorts.^16,19,20,24,33,34,38,41,48,57,61^ The number of patients per study varied widely, with large imaging-report numbers averaging over 20 000 documents, which can enable robust training. In contrast, smaller, more specialized data sets had fewer samples. Data sets normally encompass structured clinical variables, unstructured text, and multimodal combinations, where structured-only approaches commonly prioritize tabular EHR features and more text-centered narratives extracted via NLP.^16,24,26,28,32,34,37,49,50,56^

#### Modeling Approaches and Interpretability

Several NLP approaches were used, ranging from rule-based extraction to traditional ML, deep learning, and transformers. Hybrid pipelines often achieved high area under the receiver operating characteristics for classification tasks. In parallel, transformer-based models, such as BERT and XL-Net, have demonstrated strong performance, particularly on metrics like the F1-score, for complex language understanding tasks, including concept extraction from clinical text. Shapley Additive Explanations and attention mechanisms tend to show better interpretability when compared with other techniques. NLP showed high accuracy in concept extraction, but had problems with fragmented/misspelled keyword detection and non-English texts, where rule-based techniques were able to extract structured fields, and deep learning handled semantic nuances.^18,33,35–37,40,55,58,61–63^

#### Model Performance Metrics

A comparative analysis of algorithmic approaches reveals distinct performance patterns. LLM, particularly GPT-4 and Llama 3.1 405B, demonstrated high proficiency in extracting structured data from clinical notes, achieving accuracies of 93.5% to 95.1%, and processing reports in seconds, compared with manual hours. However, their performance was contingent on the quality of the documentation, and sometimes they exhibited cautious reasoning or made guesses with incomplete data. In contrast, fine-tuned BERT variants (eg, PubMedBERT, ClinicalBERT) excel in complex classification tasks, such as identifying stroke complications or cause, often achieving AUCs ≥0.90 by leveraging domain-specific pretraining to capture nuanced clinical context. Traditional Machine Learning models (eg, Extreme Gradient Boosting algorithm, Random Forests) remained highly competitive, especially for structured data prediction tasks such as readmission or mortality risk (AUCs up to 0.98), with authors noting their advantage in interpretability via Shapley Additive Explanations analysis. Crucially, the literature consistently showed that ensemble methods and multimodal fusion of structured and unstructured data outperformed single-modality baselines, though these hybrid models were also most susceptible to performance degradation (eg, ≈24% accuracy drop) in external validation, highlighting a universal challenge of generalizability.^16,18,22,26,33,34,46,48,49,57,64^

#### LLM-Specific Application

LLM demonstrated strong ability in report summarization, extraction, and decision-making support, with chatbot interfaces achieving high sensitivities. Fine-tuning local models matched GPT-4’s extraction accuracy while reducing workflow times. LLM demonstrated competitive performance in clinical tasks, with GPT-4 outperforming GPT-3.5. The key LLM applications included surgical report extraction, treatment recommendation, and NIHSS scoring. The major limitations included mathematical errors when computing volumes, hallucinations, and format inconsistencies.

The primary advantage of local LLM is maintaining privacy while developing the models.^17–19,28–30,34,43,47,65–67^

#### Multimodal Data Integration

Radiology reports provided rich information for classification and prognosis, with NLP applied techniques detecting occlusions, hemorrhages, and scores with accuracies exceeding 90%. Although the report analysis time is drastically reduced, accuracy varies depending on the rarity of the condition. Radiology reports were used in 37% of the studies, with computed tomography/magnetic resonance imaging as the most common modalities. The analysis has shown that evaluating entire documents outperforms approaches that use an arbitrary number of sentences. Multimodal integration between imaging and clinical data boosted prediction.^21,33,35,37,49,50,68^ EHRs were utilized in many papers, while combining structured tables with clinical notes. EHR is the primary data modality used (89% of studies), with discharge summaries being the most commonly utilized document. NLP augmentation has been shown to reduce missing information and improve risk score calibration; however, single-site data limitations affected most studies.^24,27,32,38,41,48,56,57,69,70^

Integrating multiple modalities enhances predictiveness, with studies combining structured EHR, unstructured text, and imaging features achieving AUCs between 7% and 21% higher than those of unimodal approaches. Temporal data integration provided valuable information. Outcome predictive models demonstrated robust discrimination but faced calibration challenges, with functional outcome and mortality models utilizing combined data. The key findings showed that multimodal methods outperformed clinical scores and imaging predictions individually, and time-series analysis of vital signs enhances intensive care unit prediction.^14,17,20–22,26,27,30,71^

### Methodological and Clinical Barriers in Stroke

#### Data Limitation

A limited data set size is one of the most frequent challenges in studies, compromising statistical power, hindering subgroup analyses, and increasing the risk of overfitting, particularly for rare conditions or underrepresented critical outcomes, such as midline shifts. Such constraints can impede the training of complex models and also reduce confidence in generalization.^16,22,25,28,29,32,34–36,42,46,72,73^ The generalizability is often restricted to a specific population group and methodological implementation, and can even be affected by the underrepresentation of ethnic minorities in training data. From the studies, models were often trained in English/German-only scenarios, with variation of different terminologies across sites, and deep reliance on single-center data, later degrading the performance and limiting the transferability across sites or documentation practices.^14,16,18,19,21,23,33,35,38,45,46,57–59,67^

#### Documentation Quality and Heterogeneity

Limitations related to data heterogeneity, gaps in documentation, inconsistent variables across sites, and ambiguous terminologies are often seen in the group of reviewer papers. Algorithm performance often relies on the quality of source documents (eg, discharge summaries), with risks of propagating errors from narrative reports that lack imaging or laboratory confirmation. On the other contrary, other challenges reported include missing critical variables (eg, cholesterol, blood pressure levels), variability in EHR content, distinct homonyms and clinical abbreviations, and feature extraction impaired by vague notes or ambiguous medical terms.^21,27,32,36–38,44,49,50,52,53,55–57,63,74^ Reliance on single, institutional-specific information introduced selection biases, limited temporal or cross-site validation, and diminished the applicability in real-world scenarios.^17,19,24,27,33,34,38,39,48,55,57,62,66,75,76^

Neurological examination documentation represents a particularly challenging source of clinical ambiguity in stroke care. Subtle linguistic qualifiers commonly used in neurological assessments (eg, mild drift, partial neglect, difficult to assess due to aphasia, or limited by poor cooperation) may not map reliably onto standardized severity scales such as the NIHSS. This limits the safety and validity of fully automated neurological severity scoring and highlights the need for clinician-in-the-loop validation when LLMs are used for neurological assessment tasks.

#### Interpretability and Clinical Acceptance

The clinical acceptance of machine learning and LLM techniques often stems from their black box nature, which makes it difficult to interpret the pathways chosen by the models to highlight certain findings. Other criticisms are related to hallucinations, misidentifications, reduced expert interpretation, potential over-triage without clear need, and wrong risk estimation.^24,28,41,65,75^ Some studies, due to limited information, overlooked dynamic physiological signals and imaging data, relying solely on textual data. This resulted in the inability to (1) read real-time changes, (2) integrate social health information, (3) interpret imaging artifacts, and (4) incorporate clinical scales.^16,17,19,40,48^ Although the fast advance of LLM approaches, translation barriers still exist—physicians need to supervise the automated recommendations due to ethical risks from aggressive or unsupported suggestions, poor patient communication, lack of regulatory certification, and undefined or limited clinician-AI interaction.^16–19,24,28,30,32,36,42,45,60,65,66,74^

#### Model Failure Modes and Linguistic Robustness

Models often show limitations regarding hallucinations, logical inference failures, nonadaptability to linguistic variations, contextual misunderstandings, excessive care causing over-triage, and error in anatomic localization.^16–19,28,30,33,41,44,45,47,54,58,65,68^ On the other hand, computational and hardware barriers can also make the implementation difficult due to the high resources that LLMs require. In addition, the lack of multimodality integration, token truncations (ie, quantization), and dependency on specific data formats can impact clinical workflows (23%).^17,20,23,26,41–43,46,51,53,63,70,72,75,77^ However, even in cases where both the model and its infrastructure are correctly set, the integration into the clinical setting can become problematic. From the issues highlighted in the studies, they commonly refer to insufficient or undermined clinical readiness, lack of external validation, absence of real-world trial problems, and performance degradation when including multicenter validation due to complete heterogeneous documentations.^29,31,32,34,37,40,47,49,50,64,69,73,76,78,79^

### Future Research Directions and Emerging Pathways

#### Validation and Deployment

The need for larger, multicenter trials to ensure stroke-related LLM and NLP models is vastly emphasized in the studies.^19,28,32,46,47^ Validating language-based systems across healthcare sites and demographics is a consensus priority, requiring multicenter testing to accurately assess real-time performance, generalizability, and address site-specific biases.^32,34,37,38,47,49,57,71^

#### Multimodal Integration

Integrating multimodalities has been shown to enhance models’ accuracy and improve clinical utility by combining structured EHRs, imaging, vitals, wearable devices, and genetic data.^48,55,57^ Studies have shown that combining lab values, social factors, and postacute metrics is ideal for tasks such as risk prediction.^21,22,26,57^ Integrating real-time EHRs is critical for adopting these models into routine care,^16,24,65^ potentially combining structured with unstructured data.^21,55,67^

#### Model Interpretability and Trust

Model trust and clinical acceptance depend on how transparent the models are to highlight not only the clinical decisions, but also the reasoning behind such decisions. Interpretability modules, such as Shapley Additive Explanations, decision trees, and error analysis, for varied prompts are vital in overcoming the black box concepts of learning methods.^25,33,38^ Addressing ethical concerns requires explainable methods, bias mitigation, and ethical frameworks,^19,25,28,34,39,60^ ideally supported by a track of decision sources, regulatory information, and comprehensive outputs.^18,29,60^

#### Workflow Integration

The translation of language models to clinical practice needs to be adaptive to live workflows, including voice interfaces and EHR-integrated dashboards.^32,76^ Prospective validation in emergency services and stroke units is quantified by time saved, safety, and resource optimization.^47,76^ Key applications include automated registry management, medical platforms,^44,47,66,76^ and workflow optimization to reduce documentation problems.^39,42,46,73^ Transfer learning from admission data can enable early intervention if adopted properly.^48^

#### Technical Optimization

The optimization of model performance for rare events involves prompt engineering, few-shot learning, and parameter fine-tuning (eg, low-rank adaptation).^16,45,47^ Adaptive prompting and reinforcement learning can mitigate hallucinations.^16,45,47^ The models can also be refined by adopting bias reduction through fine-tuning,^28,31^ addressing data imbalance,^31,73^ enhancing temporal reasoning,^66,72^ and optimizing computational resources.^41,58^

#### Ethics, Regulation, and Evaluation

An ethical framework and its subsequent regulation after Food and Drug Administration approval are crucial steps for the acceptance of language models.^17–19^ However, this requires patient-centered analysis and prevention against model hallucinations,^17,19^ along with recurrent checks to balance efficiency and speed in clinical settings.^74^ The most effective pathway to quantify real-world needs is by measuring clinician time savings,^32,35^ assessing the impact on treatment decisions,^29,73^ and evaluating patient outcomes.^32,76^ Analysis related to cost^47^ and workflow efficiency^30,73^ emphasizes metrics such as thrombolysis delays and resource allocation.^19,29,39^

#### Equity and Language Adaptation

Ensuring equity is not an easy task, because it demands extensive validation for multilanguages and diverse demographic groups, adapting to less structured text when needed.^34,38,71^ This includes, but is not limited to, multilingual model development, specialized training, and the inclusion of underrepresented populations to prevent disparities or biases.^34,38,71^ In addition, expanding the data set to capture rare comorbidities and social factors can promote fairness.^34,38,71^ The application of equity concerns can be utilized for automated coding audits, identifying rare diseases,^38,65^ cardiovascular or chronic conditions,^41,52,77^ and ontology expansion for broader applicability.^36,53^

#### Hybrid Methods and Architectural Advances

The use of hybrid methods that combine rule-based and deep learning architectures has been shown to improve efficiency.^31,33,36^ Joint learning frameworks (ie, multiple related models trained simultaneously) can enhance data extraction and stochastic sampling for rare conditions,^31,33,36^ whereas template-based synthesis is primarily used for domain adaptation.^31,33,36^ The use of hybrid methods enables rapid customization to new data types, preserves interpretability, and reduces problems such as annotation requirements.^31,33,36^ Hierarchical architecture and ensembles can also support the combination of multiple modalities.^21,23^

### Stroke-Specific Clinical Considerations

#### Acute Triage and Real-Time Decision Environments

Future LLM applications in stroke must be evaluated within real-time clinical environments, particularly prehospital and emergency department workflows. Performance metrics should extend beyond offline accuracy and include latency, reliability under time pressure, and impact on clinically meaningful benchmarks such as door-to-needle and door-to-groin times. This suggests a need for task-specific, lightweight models rather than reliance on general-purpose systems.^19,73,80^

#### Neurological Assessment and Severity Scoring

Applications involving neurological severity scoring require explicit safeguards. Future models should be designed with clinician-in-the-loop validation for tasks such as NIHSS score derivation, rather than fully automated scoring, given the linguistic ambiguity inherent in neurological examinations.^43,56^

#### Etiological Classification and Clinical Reasoning

Future research should treat ischemic stroke etiological classification as a decision-support problem rather than a diagnostic automation task. LLMs may assist in synthesizing heterogeneous clinical information to generate etiological hypotheses, but final classification must remain clinician-driven due to the high risk of misclassification and downstream treatment consequences.^79,81^

#### Multimodal Clinical Integration

Clinically relevant LLM systems will require formal integration with neuroimaging and structured clinical data. Text-only architectures are unlikely to support real-world stroke workflows, and future models should explicitly evaluate how language-based reasoning can be combined with imaging-derived features for treatment eligibility and outcome prediction.^51,73,82^

#### Longitudinal Care and Recovery Monitoring

Future applications may be most impactful in the chronic phase of stroke care, where LLMs can support longitudinal monitoring of recovery, complications, and functional outcomes through synthesis of fragmented outpatient and rehabilitation documentation.^42,67,83^

#### Workflow Integration Across the Stroke Care Pathway

NLP approaches applied to rehabilitation documentation suggest that language models can assist in standardizing and summarizing care plans across multidisciplinary teams. These integration points align with current evidence and avoid direct interference with high-risk clinical decisions.^19,42,73^

## Conclusions

In this review, we separated our analysis into 2 parts (quantitative and qualitative). For our quantitative analysis, we reported: (1) how the impact factor evolved over time for each technique; (2) which journals were commonly used for publications in this field; and the distribution between journal papers and conference proceedings. Our qualitative analysis extended longer in our document, examining: (1) motivation or purpose of each work (eg, risk prediction, treatment decision support, data extraction; (2) data set main features and key findings (eg, public versus private data, multimodal integration); (3) main limitations (eg, data quality, generalizability concerns, validation gaps); and (4) proposed future directions (eg, multicenter validation, real-time data integration, interpretability).

After our thorough evaluation of the published works, we observed a rapid growth in LLM-based publications, achieving impact factors comparable to those of early, established NLP-based studies. Journal articles featured more in-depth and exploratory analyses, whereas conference proceedings focused on delivering faster, problem-specific results. In general, papers mentioned rigorous and structured text analysis for stroke prediction (38%); LLM approaches for clinical workflow optimization were present in 23% of the studies, whereas risk prediction reached 28%. Some of the key findings include: (1) the common adoption of private data in the studies (85%), making reproduction by other groups difficult; (2) frequent need for EHR pipelines and richer annotations (89%), which can reduce the chances for smaller groups to reproduce the results without hospital partnerships; (3) common single-center design (63%), limiting generalizability. Some concerning limitations are also described: (1) external validation gaps (94%), showing the urgent need for external benchmarks and real-world deployment; (2) generalizability concerns (74%), often resulting in model hallucinations or formatting inconsistencies. Additional challenges highlighted are nontranslation to the clinical setting, small sample sizes, heterogeneity across sites, and infrastructural constraints.

From a clinical perspective, this systematic review highlights that LLMs are not yet suitable for autonomous decision-making in high-risk stroke scenarios such as acute triage, treatment selection, or etiological diagnosis. At present, their most reliable clinical value lies in lower-risk applications, including documentation support, data extraction, report summarization, and longitudinal patient monitoring. Meaningful clinical translation will require prospective validation in real-world stroke workflows, formal integration with neuroimaging and structured clinical data, and codevelopment with clinicians to ensure safety, interpretability, and accountability. Until such evidence exists, LLMs should be viewed as tools for clinical augmentation rather than replacements for expert neurological judgment.

Future directions should prioritize large-scale, multicenter validation of models to ensure applicability for diverse populations and settings (82%), including underrepresented demographic and linguistic groups. Emphasizing transparent, interpretable models for clinical decisions (23%), using techniques like Shapley Additive Explanations. Technical adjustments should focus on reducing hallucinations through prompt engineering and fine-tuning strategies (98%), as well as managing imbalances or rare-event data via approaches such as few-shot learning strategies. The clinical impact should always consider rigorous and validated outcomes, cost, and the benefits to support clinicians and increase their confidence in models (85%).

## ARTICLE INFORMATION

### Author Contributions

K.T.N. Duarte was responsible for the study concept and design, defined the review protocol, performed study selection, and conducted data analysis and interpretation. He also drafted the initial and updated manuscript versions. A.S. Sidhu and M.C. Barros contributed to the full-text review of selected studies and the curation of the final paper selection. M. Bakshi contributed to the synthesis of the main findings, the categorization of selected studies, and the creation of the figures. B. Karmur, T. Aslan, and M. AlShamrani identified the medical significance of the findings, selecting medically relevant topics of interest, and providing critical revision of the manuscript from a clinical perspective. D. Zhang and W. Qiu focused on identifying the computational contributions, refining the relevant sections, and advising on key computational methodologies and nomenclature. B.K. Menon provided overall supervision, defined core thematic groups, curated the most impactful examples, and led the critical revision of the manuscript through multiple iterations. All authors reviewed, provided feedback, and approved the first and second drafts, as well as the final manuscript version.

### Disclosures

This study did not require, nor needed, any ethical board approval or written consent from patients as it is designed as a review study of published papers. The authors report no conflicts.

## References

[1] William JP Alejandro AR Teri A Opeolu MA Nicholas CB Kyra B Jose B Michael B Bart MD. Guidelines for the early management of patients with acute ischemic stroke: a guideline for healthcare professionals from the American Heart Association/American Stroke Association. Stroke. 2018;49:e46–e110. doi: 10.1161/STR.0000000000000158 29367334 10.1161/STR.0000000000000158

[2] Turc G Bhogal P Fischer U Khatri P Lobotesis K Mazighi M Schellinger PD Toni D de Vries J White P. European Stroke Organisation (ESO)–European Society for Minimally Invasive Neurological Therapy (ESMINT) guidelines on mechanical thrombectomy in acute ischemic stroke. J NeuroInterv Surg. 2019;15:e8. doi: 10.1136/neurintsurg-2018-014569 30808653 10.1136/neurintsurg-2018-014569

[3] Carolee JW Joel S Ross A Barbara B Leora RC Steven CC Frank D Janice JE Beth F Richard LH. Guidelines for adult stroke rehabilitation and recovery: a guideline for healthcare professionals from the American Heart Association/American Stroke Association. Stroke. 2016;47: e98–e169. doi: 10.1161/STR.0000000000000098 27145936 10.1161/STR.0000000000000098

[4] Chin-Fu L Johnny H Xin X Sandhya R Victor W Michael IM Argye EH Andreia VF Max W Steven JW. Deep learning-based detection and segmentation of diffusion abnormalities in acute ischemic stroke. Commun Med. 2021;1:61. doi: 10.1038/s43856-021-00062-8 35602200 10.1038/s43856-021-00062-8 PMC9053217

[5] Ramsha A Aamna AS Naoufel W Mohamed LS. Segmentation of stroke lesions using transformer-augmented MRI analysis. Hum Brain Mapp. 2024;45:e26803. doi: 10.1002/hbm.26803 39119860 10.1002/hbm.26803 PMC11310771

[6] Nathan AS Ammad AB Muhammad W Tatsat RP Rimal HD Megan W Justin MC Adnan HS Vincent MT Elad IL. Artificial intelligence for large-vessel occlusion stroke: a systematic review. World Neurosurg. 2022;159:207.e1–220.e1. doi: 10.1016/j.wneu.2021.12.004 34896351 10.1016/j.wneu.2021.12.004 PMC9172262

[7] Gauri P Balakrishnan K Bikikumar S Aakanshrahul M Aiswariyaanna A Vindhesh D Hassan M Muhammad S. Emerging artificial intelligence-aided diagnosis and management methods for ischemic strokes and vascular occlusions: a comprehensive review. World Neurosurg. 2024;22:100303. doi: 10.1016/j.wnsx.2024.100303 10.1016/j.wnsx.2024.100303 PMC10951088 38510336

[8] McDougall CC Chan L Sachan S Guo J Sah RG Menon BK Demchuk AM Hill MD Forkert ND Esterre CD. Dynamic CTA-derived perfusion maps predict final infarct volume: the simple perfusion reconstruction algorithm. Am J Neuroradiol. 2020;41:2034–2040. doi: 10.3174/ajnr.A6783 33004342 10.3174/ajnr.A6783 PMC7658815

[9] Jelle D Anke W Soren C Robin L Maarten GL. Review of perfusion imaging in acute ischemic stroke. Stroke. 2020;51:1017–1024. doi: 10.1161/STROKEAHA.119.028337 32008460 10.1161/STROKEAHA.119.028337

[10] Jeong-Myeong C Soo-Young S Pum-Jun K Yu-Seop K Sang-Hwa L Jong-Hee S Dong-Kyu K Jae-Jun L Chulho K. Prediction of hemorrhagic transformation after ischemic stroke using machine learning. J Pers Med. 2021;11:863. doi: 10.3390/jpm11090863 34575640 10.3390/jpm11090863 PMC8470833

[11] Xuewen L Changyan X Chengming S Yiting W Jiancheng X Zhou Q. Machine learning predicts the risk of hemorrhagic transformation of acute cerebral infarction and in-hospital death. Comput Methods Programs Biomed. 2023;237:107582. doi: 10.1016/j.cmpb.2023.107582. 37156021 10.1016/j.cmpb.2023.107582

[12] Qingqing L Hongyi C Junyan F Xiaodong Z Yiren X Yuning P. Automatic collateral quantification in acute ischemic stroke using u2-net. Front Neurol. 2025:16:1502382. doi: 10.3389/fneur.2025.1502382 40421138 10.3389/fneur.2025.1502382 PMC12104720

[13] Valeria CS Gabriel AS Bernardo Corr´ea de AT Viviane de HF Zˆetola´Marcos CL. Automated evaluation of collateral circulation for outcome prediction in acute ischemic stroke. J Stroke Cerebrovasc Dis. 2024;33:107584. doi: 10.1016/j.jstrokecerebrovasdis.2024.107584 38246577 10.1016/j.jstrokecerebrovasdis.2024.107584

[14] Arlene C Emma D Claire G Richard T Andreas G Huayu Z Patrick S Alison QO Liam L Michael W, et al. Understanding the performance and reliability of NLP tools: a comparison of four NLP tools predicting stroke phenotypes in radiology reports. Front Digit Health. 2023;5:1184919. doi: 10.3389/fdgth.2023.1184919 37840686 10.3389/fdgth.2023.1184919 PMC10569314

[15] Barbara K O. Pearl B David B Mark T John B Stephen L. Systematic literature reviews in software engineering – a systematic literature review. Inf Softw Technol. 2009;51:7–15.

[16] Mariyam A Shams K Noman A Mira S Yasin M. A smart recommender system for stroke risk assessment with an integrated strokebot. J Med Biol Eng. 2024;44:799–808. doi: 10.1007/s40846-024-00922-3

[17] Tse CC Mitchell WC Jorie S Alyssa S Laila K Emily K Khoa N Evan M Aaron SD Arthur W. Assessing the clinical reasoning of ChatGPT for mechanical thrombectomy in patients with stroke. J Neurointerv Surg. 2024;16:253–260. doi: 10.1136/jnis-2023-021163 38184368 10.1136/jnis-2023-021163

[18] Jonathan K Robert H Andra-Iza I Philipp F Johannes B David Z Kai RL Oezguer AO Simon L Michael S, et al. Large language models-supported thrombectomy decision-making in acute ischemic stroke based on radiology reports: feasibility qualitative study. J Med Internet Res. 2025;27:e48328. doi: 10.2196/48328 39946168 10.2196/48328 PMC11888093

[19] Merve Y Emine S. Compliance evaluation with ChatGPT for diagnosis and treatment in patients brought to the ED with a preliminary diagnosis of stroke. Prehosp Emerg Care. 2025;29:243–251. doi: 10.1080/10903127.2025.2475513 40036089 10.1080/10903127.2025.2475513

[20] Qizhang F, Jiayi Y, Forhan BE, Karim H, Xia H, and Zhe H. Can Attention be used to explain EHR-based mortality prediction tasks: a case study on hemorrhagic stroke. The 14th ACM International Conference on Bioinformatics, Computational Biology, and Health Informatics (ACM BCB 2023). September 2023. Houston, TX. pp. 1–6. doi: 10.1145/3584371.3613002 10.1145/3584371.3613002 PMC11262850 39040997

[21] Ruixuan H Jundong L Tsz KW Damrongrat S Yat Ming PW Asmir V Chi WW Kei HKC. Stroke mortality prediction based on ensemble learning and the combination of structured and textual data. Comput Biol Med. 2023;155:106176. doi: 10.1016/j.compbiomed.2022.106176 36805232 10.1016/j.compbiomed.2022.106176

[22] Ling-Chien H Ying-Ying S Jui-Ming S Wan-Ting H Sheng-Feng S. Clinical narratives as a predictor for prognosticating functional outcomes after intracerebral hemorrhage. J Neurol Sci. 2023;453:120807. doi: 10.1016/j.jns.2023.120807 37717279 10.1016/j.jns.2023.120807

[23] Yikuan L Mohammad M Gholamreza SK Shishir R Abdelaali H Dexter C Thomas L Kazem R. Hi-BEHRT: hierarchical transformer-based model for accurate prediction of clinical events using multimodal longitudinal electronic health records. IEEE J Biomed Health Inf. 2023;27:1106–1117. doi: 10.1109/JBHI.2022.3224727 10.1109/JBHI.2022.3224727 PMC7615082 36427286

[24] Christina ML Ravi G Elissa O Andrew MN Jane LH Shyam P. Prediction of 30-day readmission after stroke using machine learning and natural language processing. Front Neurol. 2021;12:649521. doi: 10.3389/fneur.2021.649521 34326805 10.3389/fneur.2021.649521 PMC8315788

[25] Lv J Mengmeng Z Yujie F Mengshuang C Binjie C Zhiyuan X Xianliang Y Shuqun H Ningjun Z. An interpretable machine learning approach for predicting 30-day readmission after stroke. Int J Med Inform. 2023;174:105050. doi: 10.1016/j.ijmedinf.2023.10505 36965404 10.1016/j.ijmedinf.2023.105050

[26] Danqing M Meng W Xiang A Zongqing Q Qin Y. Transformer-based classification outcome prediction for multimodal stroke treatment. In: 2024 IEEE 2nd International Conference on Sensors, Electronics and Computer Engineering (ICSECE). IEEE; 2024;383–386.

[27] Sheng-Feng S Kuan-Lin S Ru-Chiou P Pei-Ju L Ya-Han H. Automated risk assessment of newly detected atrial fibrillation poststroke from electronic health record data using machine learning and natural language processing. Front Cardiovasc Med. 2022;9:941237. doi: 10.3389/fcvm.2022.941237 35966534 10.3389/fcvm.2022.941237 PMC9372298

[28] Gal BH Adi B Haggai E Livnat B Yiftach B Avinoah I Eyal K. AI in the ED: assessing the efficacy of GPT models vs. physicians in medical score calculation. Am J Emerg Med. 2024;79:161–166. doi: 10.1016/j.ajem.2024.02.016 38447503 10.1016/j.ajem.2024.02.016

[29] Bing YC Fares A Marco G Ken U Samer A Scott R Sidonie I Eric A Andrew R Muhammad SH. Automated identification of stroke thrombolysis contraindications from synthetic clinical notes: a proof-of-concept study. Cerebrovasc Dis Extra. 2025;15:130–136. doi: 10.1159/000545317 40096831 10.1159/000545317 PMC12021381

[30] Amit HS Mark K Michal CS Shlomi P Dvir A Shahar S. GPT-4 as a clinical decision support tool in ischemic stroke management: evaluation study. JMIR AI. 2025;4:e60391. doi: 10.2196/60391. 40053715 10.2196/60391 PMC11928773

[31] Davide B Akanksha A Umberto P Giovanni A Simona M. Development of a natural language processing (NLP) model to automatically extract clinical data from electronic health records: results from an Italian comprehensive stroke center. Int J Med Inform. 2024;192:105626. doi: 10.1016/j.ijmedinf.2024.105626 39321491 10.1016/j.ijmedinf.2024.105626

[32] Rudy G Benjamin C Brandon S Andrew ECB Shrirajh S Sarah H Joshua K Aashray G Sheryn T W. TK. Large language models can effectively extract stroke and reperfusion audit data from medical free-text discharge summaries. J Clin Neurosci. 2024;129:110847. doi: 10.1016/j.jocn.2024.110847 39305548 10.1016/j.jocn.2024.110847

[33] Dane G Paulo PP Olivier M Rebecca ET Amy YXY Zhongyu AL Muhammad M Chloe PP Richard IA. Rule-based natural language processing for automation of stroke data extraction: a validation study. Neuroradiology. 2022;64:2357–2362. doi: 10.1007/s00234-022-03029-1 35913525 10.1007/s00234-022-03029-1

[34] Nils CL Johannes K Barbara DW Moritz W Zeynep B Felix JB Hanna Z Alexander R Philipp V Franziska D. Llama 3.1 405B is comparable to GPT-4 for extraction of data from thrombectomy reports-a step towards secure data extraction. Clin Neuroradiol. 2025;35:495–510. doi: 10.1007/s00062-025-01500-z 39998651 10.1007/s00062-025-01500-z PMC12454497

[35] Matthew M Agni O Michael C Hanife S Oluwafemi B Maria T Kyriakos V Georgia F Nina MF Jack K. Natural language processing of radiology reports to detect complications of ischemic stroke. Neurocrit Care. 2022;37(Suppl 2):291–302. doi: 10.1007/s12028-022-01513-3 35534660 10.1007/s12028-022-01513-3 PMC9986939

[36] Sonish S Fengyi G Parker D Bayan A Shyam V Allyn B Yanshan W. Mining clinical notes for physical rehabilitation exercise information: natural language processing algorithm development and validation study. JMIR Med Inform. 2024;12:e52289. doi: 10.2196/52289 38568736 10.2196/52289 PMC11024747

[37] Amy Y XY Zhongyu AL Chloe PP Kaitlyn L Moira KK Richard IA Muhammad M. Automating stroke data extraction from free-text radiology reports using natural language processing: instrument validation study. JMIR Med Inform. 2021;9:e24381. doi: 10.2196/24381 33944791 10.2196/24381 PMC8132979

[38] Stephen B Sam G Simon K Jim J Timothy K. Improving the accuracy of stroke clinical coding with open-source software and natural language processing. J Clin Neurosci. 2021;94:233–236. doi: 10.1016/j.jocn.2021.10.024 34863443 10.1016/j.jocn.2021.10.024

[39] Yeshwant RC Shourya M Benjamin L Timothy LC Gunvant RC Thienkhai V Youngho S Jared N Jae HS. Development and web deployment of an automated neuroradiology MRI protocoling tool with natural language processing. BMC Med Inform Decis Mak. 2021;21:213. doi: 10.1186/s12911-021-01574-y 34253196 10.1186/s12911-021-01574-y PMC8276477

[40] Zhanzhong G Xiangjian H Ping Y Wenjing J Xiguang Y Gang P Penghui H Shiyan C Hongjie C Yiguang L. Automatic quantitative stroke severity assessment based on Chinese clinical named entity recognition with domain-adaptive pre-trained large language model. Artif Intell Med. 2024;150;102822. doi: 10.1016/j.artmed.2024.102822 38553162 10.1016/j.artmed.2024.102822

[41] Jaeyoung K Sihyeon L Hyeon J Keon-Joo L Hee-Joon B Bohyoung K Jinwook S. PhenoFlow: a HumanLLM DRIVEN visual analytics system for exploring large and complex stroke datasets. IEEE Trans Vis Comput Graph. 2025;31:470–480. doi: 10.1109/TVCG.2024.3456215 39316495 10.1109/TVCG.2024.3456215

[42] Hunter O Courtney ES Haneef AM Minmei S Alexandra EH Leming Z Elizabeth RS Yanshan W. Automated fidelity assessment for strategy training in inpatient rehabilitation using natural language processing. AMIA Jt Summits Transl Sci Proc. 2023;438–447. doi: 10.48550/arXiv.2209.06727 37350902 PMC10283102

[43] Zhanzhong G Wenjing J Massimo P Ping Y. Empowering large language models for automated clinical assessment with generation-augmented retrieval and hierarchical chain-of-thought. Artif Intell Med. 2025;162:103078. doi: 10.1016/j.artmed.2025.103078 39978047 10.1016/j.artmed.2025.103078

[44] Jung-Hyun L Eunhee C Robert MD William WL. GPT-4 performance for neurologic localization. Neurol-Clin Pract. 2024;14:e200293. doi: 10.1212/CPJ.0000000000200293 38596779 10.1212/CPJ.0000000000200293 PMC11003355

[45] Xiaowei S Jiayi W Feifei H Wei Y Weizhi M Jian W. Stroke diagnosis and prediction tool using ChatGLM: development and validation study. J Med Internet Res. 2025;27:e67010. doi: 10.2196/67010 40009850 10.2196/67010 PMC11904371

[46] Mengfei W Jianyong W Yao Z Lisong D Bicong Y Yueqi Z Xiaoer W Yidong J Yuehua L. Precision structuring of free-text surgical record for enhanced stroke management: a comparative evaluation of large language models. J Multidiscip Healthc. 2024;17:5163–5175. doi: 10.2147/JMDH.S486449 39558925 10.2147/JMDH.S486449 PMC11572044

[47] Xinhao W Shisheng Y Jinwen F Kaiyan F Heng Y Hao L. Performance of ChatGPT on prehospital acute ischemic stroke and large vessel occlusion (LVO) stroke screening. Digit Health. 2024;10:20552076241297127. doi: 10.1177/20552076241297127. 39507012 10.1177/20552076241297127 PMC11539183

[48] Ho-Joon L Lee HS Lauren HS Hooman K Adam de H Ashby CT Kevin NS Smita K Cynthia B Hongyu Z, et al. StrokeClassifier: ischemic stroke etiology classification by ensemble consensus modeling using electronic health records. NPJ Digital Med. 2024;7:130. doi: 10.1038/s41746-024-01120-w 10.1038/s41746-024-01120-w PMC11101464 38760474

[49] Li MD Lang M Deng F Chang K Buch K Rincon S Mehan WA Leslie-Mazwi TM Kalpathy-Cramer J. Analysis of stroke detection during the COVID-19 pandemic using natural language processing of radiology reports. AJNR Am J Neuroradiol. 2021;42:429–434. doi: 10.3174/ajnr.A6961 33334851 10.3174/ajnr.A6961 PMC7959438

[50] Charlene JO Agni O Rebecca Z Francois Pierre MC Meghan H Liang M Darian F Oluwafemi B Matthew M Margaret M. Machine learning and natural language processing methods to identify ischemic stroke, acuity and location from radiology reports. PLoS One. 2020;15:e0234908.doi: 10.1371/journal.pone.0234908 32559211 10.1371/journal.pone.0234908 PMC7304623

[51] Xiaowei X Qin L Lingling D Chunjuan W Meng W Zixiao L Jiao L. Identifying stroke diagnosis-related features from medical imaging reports to improve clinical decision-making support. BMC Med Inform Decis Mak. 2022;22:275. doi: 10.1186/s12911-022-02012-3 36266650 10.1186/s12911-022-02012-3 PMC9583470

[52] Audrey Y Sam K Anahita D Hwang S Meet G Ryan U Danielle M. Relation detection to identify stroke assertions from clinical notes using natural language processing. 2024;310:619–623. doi: 10.3233/SHTI231039 10.3233/SHTI231039 38269883

[53] Zhanzhong G Xiguang Y Wenjing J Chengpei X Ping Y Xiangjian H Hongjie C Yiguang L. StrokePEO: construction of a clinical ontology for physical examination of stroke. In: 2022 9th International Conference on Digital Home (ICDH). IEEE; 2022;218–223.

[54] Soonhyun K Jaehak Y Sejin P Jong-Arm J Cheol-Sig P. Stroke medical ontology QA system for processing medical queries in natural language form. In: 12TH International Conference on ICT Convergence (ICTC 2021): Beyond the Pandemic Era With ICT Convergence Innovation. IEEE; 2021;1649–1654.

[55] Adam NB David WB Curtis G Olivia LH Daniel M Orly L Wanda YW Nicholas C Jon H Deepak LB. Natural language processing for the assessment of cardiovascular disease comorbidities: the cardio-canary comorbidity project. Clin Cardiol. 2021;44:1296–1304. doi: 10.1002/clc.23687 34347314 10.1002/clc.23687 PMC8428009

[56] Ling-Chien H Ying-Ying S Jui-Ming S Wan-Ting H Sheng-Feng S. Assessing stroke severity using electronic health record data: a machine learning approach. J Neurol Sci. 2023;20:8. doi: 10.1186/s12911-019-1010-x 10.1186/s12911-019-1010-x PMC6950922 31914991

[57] Shaan K Christopher R Lia XH Pulkit S Gopal S Samuel FF Paolo DA Nathaniel D Jonathan WC Ashby CT. Cohort design and natural language processing to reduce bias in electronic health records research. NPJ Digital Med. 2022;5:47. doi: 10.1038/s41746-022-00590-0 10.1038/s41746-022-00590-0 PMC8993873 35396454

[58] Ching-Heng L Kai-Cheng H Chih-Kuang L Tsong-Hai L Ching-Sen S Yang CF. Accurately identifying cerebroarterial stenosis from angiography reports using natural language processing approaches. Diagnostics. 2022;12:1882. doi: 10.3390/diagnostics12081882 36010232 10.3390/diagnostics12081882 PMC9406429

[59] Yiqing Z Sunyang F Suzette JB Paul AD Alanna MC Veronique LR Hongfang L Nicholas BL. Natural language processing and machine learning for identifying incident stroke from electronic health records: algorithm development and validation. J Med Internet Res. 2021;23;e22951. doi: 10.2196/22951 33683212 10.2196/22951 PMC7985804

[60] Jonathan YY Soheil S Edmund H Linda SM Jennifer SR Jeffrey S Antonio T Warren W Mark IL. Accuracy of prospective assessments of 4 large language model chatbot responses to patient questions about emergency care: experimental comparative study. J Med Internet Res. 2024;26:e60291. doi: 10.2196/60291 39496149 10.2196/60291 PMC11574488

[61] Lin Y Xiaoshuo H Jiayang W Xin Y Lingling D Zixiao L Jiao L. Identifying stroke-related quantified evidence from electronic health records in real-world studies. Artif Intell Med. 2023;140:102552. doi: 10.1016/j.artmed.2023.102552 37210153 10.1016/j.artmed.2023.102552

[62] Marta F Kaileigh G Niels T Aditya G Brandon Westover M Aneesh BS Sahar FZ. Automated extraction of post-stroke functional outcomes from unstructured electronic health records. Eur Stroke J. 2025;10:829–836. doi: 10.1177/23969873251314340 39838914 10.1177/23969873251314340 PMC11752148

[63] Patrick S Hannah W Eunsoo P Maciej P Hamish MK Keith WM David HB Alison QO. Templated text synthesis for expert-guided multi-label extraction from radiology reports. Mach Learn Knowl Extr. 2021;3:299–317. doi: 10.3390/make3020015

[64] Enshuo H Abdulaziz TB Thomas P Alan PP Gavin WB Jonika T Farhaan SV. Extraction of radiological characteristics from free-text imaging reports using natural language processing among patients with ischemic and hemorrhagic stroke: algorithm development and validation. JMIR AI. 2023;2:e42884. doi: 10.2196/42884 38875556 10.2196/42884 PMC11041442

[65] Anna KF Kai Z Tia SL Xiaoqian J Stuart MF. Generative pre-trained transformer for pediatric stroke research: a pilot study. Pediatr Neurol. 2024;160:54–59. doi: 10.1016/j.pediatrneurol.2024.07.001 39191085 10.1016/j.pediatrneurol.2024.07.001

[66] Nils CL Franziska D Isabella CW Hanna Z Alexander R Jakob NK Daniel P Else K. Data extraction from free-text reports on mechanical thrombectomy in acute ischemic stroke using ChatGPT: a retrospective analysis. Radiology. 2024;311:e232741. doi: 10.1148/radiol.232741 38625006 10.1148/radiol.232741

[67] Ching-Heng L Kai-Cheng H Chih-Kuang L Tsong-Hai L Chia-Wei L Jiann-Der L Tsung P Ching-Sen S Yang CF. A disease-specific language representation model for cerebrovascular disease research. Comput Methods Programs Biomed. 2021;211:106446. doi: 10.1016/j.cmpb.2021.106446 34627022 10.1016/j.cmpb.2021.106446 PMC8551061

[68] Tak SH Yu SK Jeong MC Yeong SJ Soo YS Jun HL Jin PJ Chulho K. Prediction of stroke outcome using natural language processing-based machine learning of radiology report of brain MRI. J Personalized Med. 2020;10:286. doi: 10.3390/jpm10040286 10.3390/jpm10040286 PMC7766032 33339385

[69] Peter LE Sarah M Jack M Christopher C Sylvester S Shyamashree S Gary B Marcia W Kimberly N Joann T. Using artificial intelligence with natural language processing to combine electronic health record’s structured and free text data to identify nonvalvular atrial fibrillation to decrease strokes and death: evaluation and case-control Study. J Med Internet Res. 2021;23:e28946. doi: 10.2196/28946 34751659 10.2196/28946 PMC8663460

[70] Sheng-Feng S Chih-Hao C Ru-Chiou P Ya-Han H Jiann-Shing J. Natural language processing enhances prediction of functional outcome after acute ischemic stroke. J Am Heart Assoc. 2021;10:e023486. doi: 10.1161/JAHA.121.023486 34796719 10.1161/JAHA.121.023486 PMC9075227

[71] Sheng-Feng S Cheng-Yang H Ya-Han H. Early prediction of functional outcomes after acute ischemic stroke using unstructured clinical text: retrospective cohort study. JMIR Med Inform. 2022;10:e29806. doi: 10.2196/29806 35175201 10.2196/29806 PMC8895286

[72] Everton FB Luiz SO Alceu de SB. Predicting hospitalization with LLMs from health insurance data. Med Biol Eng Comput. 2025;63:1215–1226. doi: 10.1007/s11517-024-03251-4 39695069 10.1007/s11517-024-03251-4

[73] Chin-Fu L Zhao Y Vivek Y Richard L Vitor F Max W Steven JW Gregory WA Stephen MD James CG; STIR and VISTA Imaging Investigators. Automatic comprehensive radiological reports for clinical acute stroke MRIs. Commun Med. 2023;3:95. doi: 10.1038/s43856-023-00327-4 37430103 10.1038/s43856-023-00327-4 PMC10333348

[74] Antonio D Aurora Z Mauro G Pierandrea M Maria EL Elena A Andrea F Simona M Alice GL Sabino L. Word2vec word embedding-based artificial intelligence model in the triage of patients with suspected diagnosis of major ischemic stroke: a feasibility study. Int J Environ Res Public Health. 2022;19:15295. doi: 10.3390/ijerph192215295 36430014 10.3390/ijerph192215295 PMC9691077

[75] David MK Lester YL Yichen Z Patrick HL David FK Jason N Sunyang F Eric JP Chengyi Z Hongfang L Wansu C. Association of incidentally discovered covert cerebrovascular disease identified using natural language processing and future dementia. J Am Heart Assoc. 2023;12:e027672. doi: 10.1161/JAHA.122.027672 36565208 10.1161/JAHA.122.027672 PMC9973577

[76] Anoop M Zahra P Christopher TR William JM Jungwha L Bruce A Ohad P Scott JM Jane LH Shyam P. Improving prehospital stroke diagnosis using natural language processing of paramedic reports. Stroke. 2021;52:2676–2679. doi: 10.1161/STROKEAHA.120.033580 34162217 10.1161/STROKEAHA.120.033580 PMC8514137

[77] Yifan G Hongying Z Hongyang C Lijuan Z Kunli Z. A BART-based study of entity-relationship extraction for electronic medical records of cardiovascular diseases. In: Xu H Chen Q Lin H Wu F Liu L Tang B Hao T Huang Z, eds. HEALTH Information Processing, Chip 2023, Volume 1993 of Communications in Computer and Information Science. Springer; 2023;82–97.

[78] David MK Lester YL Yichen Z Patrick HL David FK Jason N Sunyang F Chengyi Z Hongfang L Wansu C. Association of silent cerebrovascular disease identified using natural language processing and future ischemic stroke. Neurology. 2021;97:E1313–E1321. doi: 10.1212/WNL.0000000000012602 34376505 10.1212/WNL.0000000000012602 PMC8480402

[79] Sheng-Feng S Chia-Yi L Ya-Han H. EMR-based phenotyping of ischemic stroke using supervised machine learning and text mining techniques. IEEE J Biomed Health Inf. 2020;24:2922–2931. doi: 10.1109/JBHI.2020.2976931 10.1109/JBHI.2020.2976931 32142458

[80] Ho-Jung K Dogeun P Jae-Jun L Jin-Pyeong J Dong-Ok W. A proposed LLM-based supported treatment framework for intracerebral hemorrhage. In: 2025 IEEE International Conference on Consumer Electronics (ICCE). IEEE; 2025;1–4.

[81] Xiaowei X Qin L Zixiao L Jiao L. Developing an interpretable etiology classification model for ischemic stroke based on Chinese clinical practice guideline. In: 2021 IEEE 9th International Conference on Healthcare Informatics (ICHI). IEEE; 2021;517–518.

[82] Luke M Peter K Jigar P Jay A Min Z Nathan B Kenneth W. A Comparative evaluation of large language model utility in neuroimaging clinical decision support. J Imaging Inform Med. 2025;38:2294. doi: 10.1007/s10278-024-01161-3 39508992 10.1007/s10278-024-01161-3 PMC12343430

[83] Montserrat M Santiago P Noemi T Monica M Paula O Consuelo T Marta C Pedro L Marta L Carmen M. Botulinum toxin type A (BoNT-A) use for post-stroke spasticity: a multicenter study using natural language processing and machine learning. Toxins. 2024;16:340. doi: 10.3390/toxins16080340 39195750 10.3390/toxins16080340 PMC11359065

